# Recombinant mannan-binding lectin magnetic beads increase pathogen detection in immunocompromised patients

**DOI:** 10.1007/s00253-024-13019-3

**Published:** 2024-02-03

**Authors:** Chen Xiao-Ping, Zheng Hao, Feng Ru-Li, Lu Jin-Xing, Dong Yu-Jun, Liang Ze-Yin

**Affiliations:** 1 National Institute for Communicable Disease Control and Prevention, Chinese Center for Disease Control and Prevention, Beijing, China; 2 Clinical Laboratory of Peking University First Hospital, XiShiKu Street 8, XiCheng District, Beijing, 86-10-83572211 China; 3 Department of Hematology, Peking University First Hospital, XiShiKu Street 8, XiCheng District, Beijing, 86-10-83572211 China

**Keywords:** Immunocompromised patient, M1 beads, Pathogen detection

## Abstract

**Abstract:**

The microbiological diagnosis of infection for hematological malignancy patients receiving chemotherapy or allogeneic hematopoietic stem cell transplantation (allo-HSCT) patients relies primarily on standard microbial culture, especially blood culture, which has many shortcomings, such as having low positive rates, being time-consuming and having a limited pathogenic spectrum. In this prospective observational self-controlled test accuracy study, blood, cerebrospinal fluid (CSF), and bronchoalveolar lavage fluid (BALF) samples were collected from chemotherapy or allo-HSCT patients with clinical symptoms of infections who were hospitalized at Peking University First Hospital. Possible pathogens were detected by the method based on recombinant mannan-binding lectin (MBL) magnetic bead enrichment (M1 method) and simultaneously by a standard method. The analytical sensitivity of M1 method was close to that of standard culture method. Besides, the turn-around time of M1-method was significantly shorter than that of standard culture method. Moreover, the M1 method also added diagnostic value through the detection of some clinically relevant microbes missed by the standard method. M1 method could significantly increase the detection efficiency of pathogens (including bacteria and fungi) in immunocompromised patients.

**Key points:**

• *The detection results of M1-method had a high coincidence rate with that of standard method*

• *M1 method detected many pathogens which had not been found by standard clinic method*

**Supplementary Information:**

The online version contains supplementary material available at 10.1007/s00253-024-13019-3.

## Introduction

For patients with hematological malignancies such as acute leukemia (AL), lymphoma, and multiple myeloma (MM), serial chemotherapy and autologous or allo-HSCT are life-saving therapies. Due to the reconstitution of a new immune system, the presence of neutropenia, the use of immunosuppressive drugs and graft versus host disease (GvHD), infections of the blood, lower respiratory tract, or CSF is usually common in these patients (Piñana et al. 2019; Zhang et al. 2021). Bacterial infections are the leading cause of sepsis and septic shock in these patients, whereas infections with fungal pathogens, including *Candida* spp. and *Aspergillus* spp. are becoming increasingly frequent in recent years and are usually lethal in severely immunocompromised patients (Ogura et al. 2021; Piñana et al. 2019). In addition to the complex and multifactorial process of chemotherapy or allo-HSCT, the clinical characteristics of these patients are further complicated by multi-pathogen infections. Usually, infections are the most common and significant cause of mortality and morbidity in patients with hematological malignancies (Kanda et al. 2013; Sahin et al. 2016). Thus, identifying whether there is infection and which pathogen is present are very important for the timely initiation of appropriate antimicrobial treatment, avoidance of antibiotic abuse and reduction of morbidity and mortality in these patients.

At present, because of its high specificity in bacterial species identification, standard culture, especially clinical blood culture, is still the gold standard for etiological infection diagnosis. However, clinical blood culture has many limitations, such as substantial time delay and low sensitivity, especially for slow-growing and fastidious organisms (Dubourg et al. 2019). A variety of novel methods have emerged over the past decade to overcome the limitations of clinical blood culture, including PCR, microarray and mNGS. However, there is still no widely accepted method (Peri et al. 2022).

Recombinant human mannan-binding lectin (rhMBL), which has human IgG1 Fc in N terminal and MBL carbohydrate recognition domain (CRD) in C terminal, had been used to detect pathogens previously. With its function of enriching a broad range of pathogens, the detection methods applying rhMBL were more time-saving and of higher sensitivity (Bicart-See et al. 2016; Cartwright et al. 2016; Kang et al. 2014; Kite et al. 2022). No study of detecting various pathogens in immunocompromised patients with rhMBL was performed. In our previous study, we also developed a type of magnetic beads (M1 beads) coated with a rhMBL (Chen et al. 2020). Although MBL was reported to be able to bind a variety of microorganisms, including bacteria and fungi, its binding ability was variable according to different bacterial species and even strains (Neth et al. 2000). In this study, M1 bead enrichment was found to facilitate almost all bacteria and fungi pathogen detection by detecting bacterial DNA using qPCR, even when the bacteria were not recoverable by culture.

## Materials and methods

### Patient enrollment and sample collection

From Aug 2021 to Aug 2022, hospitalized hematological malignancy patients with temperatures greater than 38 °C were included in the study at Peking University First Hospital. Patients with the following criteria were also enrolled for BALF collection: patients with clinically suspected pneumonia or those with significant imaging findings by X-ray or CT. CSF was collected from patients with symptoms of suspected central nervous system infection.

Skin disinfection was applied twice with ethanol (75%) or isopropanol (70%). Samples were drawn by an experienced nurse wearing sterile gloves. Venipuncture was used to draw blood samples into sterile BD Vacutainer Lithium Heparin tubes (BD Diagnostics, USA). Samples for M1 method were collected immediately after drawing for standard blood culture without changing blood taking needle. CSF samples were collected by lumbar puncture and gravity drip into a polypropylene tube (15 ml, Greiner Bio-One188271, Fisher Scientific, Goteborg, Sweden). Small volume bronchial washings (BALF) were performed with fiberoptic bronchoscopy by instilling 20 ml of normal saline via the bronchoscope and aspirated into suction traps. Before being sent for assays, all the samples were stored at 4 °C for no more than 72 h.

Along with the standard clinical reference method, the M1 method (see below) was performed on whole blood and CSF and BALF specimens. Additionally, some CSF and BALF samples were also examined with mNGS (Jiangsu Xiansheng Medical Diagnosis Co., Ltd.) with the consent of the patients.

In this study, an episode was defined as each separate case of clinically suspected infection treated with antibiotics. For each episode, two sets of blood cultures from two different puncture sites were collected for standard procedure. In order to minimize harm to patients, only one set of detection with the M1 method was performed.

### Diagram of the M1 method compared with the standard method

**Fig. 1:**
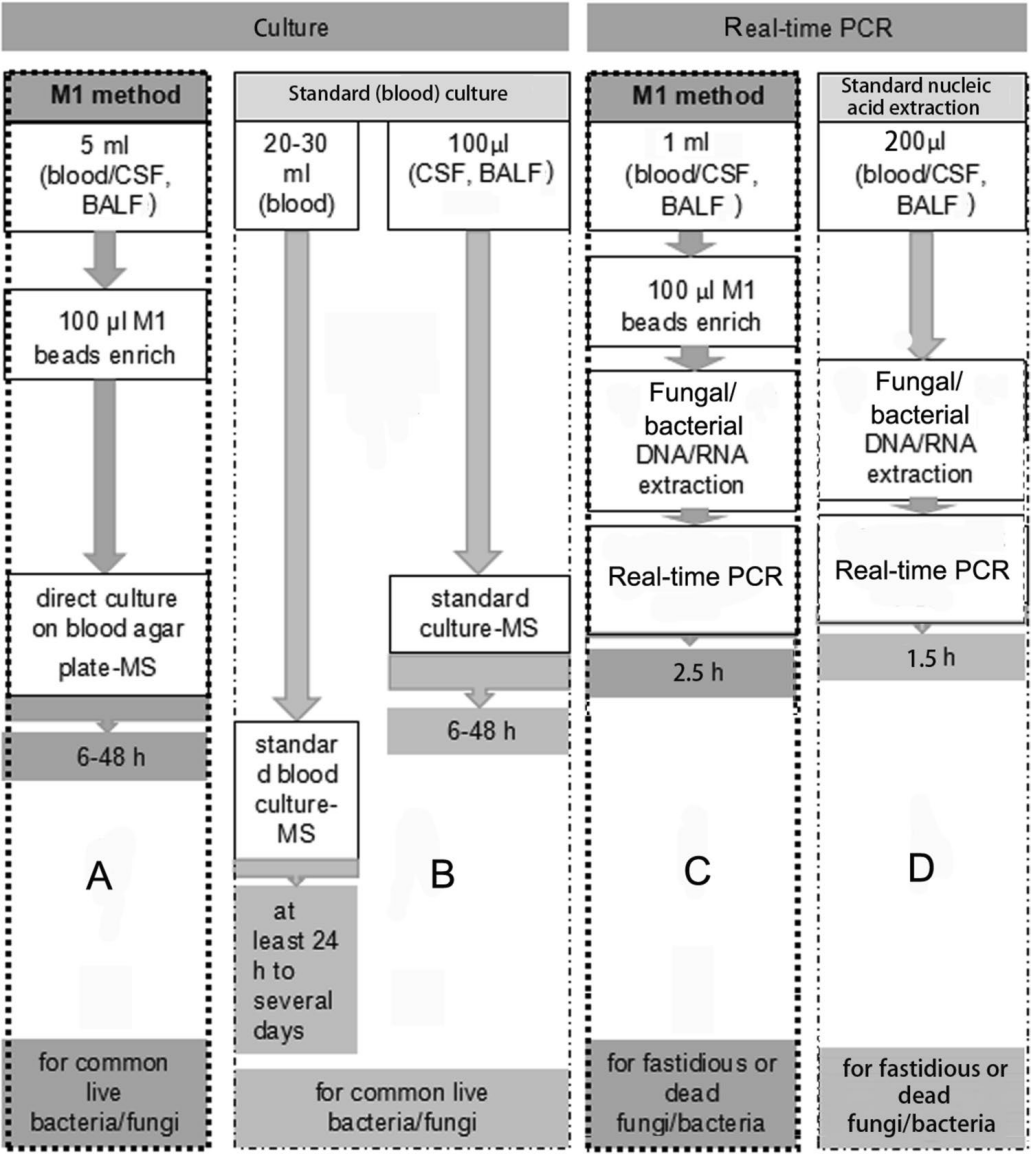
Profile of the detection workflows for the M1 method and the clinical method

The procedures for the M1 method and the standard method are shown in Fig. 1. In brief, with the M1 method, samples were treated with 100 μl of M1 beads, and direct culture (panel A) or nucleic acid extraction and real-time PCR (panel C) were performed, as described below. With the standard method, standard (blood) cultures (panel B) were routinely performed in clinical labs, and standard real-time PCR (panel D) was performed, as described below.

### M1-DC and species identification by MS

M1 bead enrichment and direct culture (M1-DC) were conducted, as described previously (Chen et al. 2020). The microbe-bound beads were then resuspended in 200 μl of PBS, plated on blood agar plates and cultured in an aerobic incubator (37 °C, 5% CO_2_) for at least 14 days. Negative controls consisting of 200 μl of M1 beads were also plated and cultured simultaneously. The result was regarded as positive if one or more microorganisms grew on the plate, while the negative control had no colonies. For direct culture using the M1 method, microorganisms were defined as contaminants and excluded if they grew after 14 days, except for *Mycobacterium*. Furthermore, to survey bead contamination, 100 μl per ml of protein A beads were removed for culture, and no microorganisms grew during the study. Typing and definite species identification with MALDI-TOF MS was performed on a Microflex LT mass spectrometer (Bruker Daltonics, USA) with BioTyper software v2.0 using default parameter settings. Spectral scores above 2.0 were used as cutoffs for correct identification.

### M1-real-time PCR method (M1-qPCR)

#### M1 bead enrichment

M1 bead enrichment was conducted as described previously (Chen et al. 2020). The following procedures were performed in a biosafety cabinet. Before the experiments, all the materials, including sample tubes, were sterilized with UV light in the cabinet for 30 min. Approximately 5 ml of blood, CSF, or BALF was collected for direct culture. In brief, PBS was added to a final volume of 12 ml with 10 mM CaCl_2_. Next, 100 μl of M1 beads was added to the sample. Negative controls consisting of 12 ml of PBS without sample were treated simultaneously. The samples were then mixed by inversion on a rotor for 45–60 min at room temperature. After incubation, the sample tubes were magnetized for 5 min on a magnetic rack. The supernatant was discarded without disturbing the beads. Approximately 1 ml of blood, CSF, or BALF was collected for bacterial/fungal nucleic acid detection. The microbe-bound beads were resuspended in 200 μl of PBS.

#### Pathogen nucleic acid extraction and detection

Nucleic acid was extracted with an OMEGA Yeast DNA Kit (OMEGA Biotech, Guangzhou, China) according to the manufacturer’s protocol. Negative controls of M1 beads were also applied for simultaneous nucleic acid extraction. Real-time PCR was performed to detect pathogen. The total reaction volume was 20 μl containing 2 μl extracted DNA, 10 μl of Probe qPCR mix (TaKaRa Bio), 0.2 μM of primers, and 0.4 μM of probe. The PCR was conducted under conditions of 95 °C for 30 s, 40 cycles of 95 °C for 5 s, and 60 °C for 30 s at last. The real-time PCR primers and probes for bacteria (*Mycobacterium tuberculosis*, non-tuberculosis mycobacteria, *Mycoplasma pneumoniae*), *Pneumocystis jirovecii* and fungi (yeast and filamentous fungi) are listed in Supplementary Table 1. Positive results were considered for those samples for which the *CT* values were lower than 38, while negative controls were higher than 38.

To confirm the bacterial or fungal colonies isolated by the M1 method, nucleic acid extracted with M1 bead enrichment was further analyzed with real-time PCR or amplified with specific primer pairs (Tables S1 and S2).

#### Standard method-clinic (blood) culture (S-BC) and MS

The standard blood cultures were analyzed using the semi-automated blood culture system BACTEC (BD Diagnostics, Sparks, MD, USA) according to laboratory-defined standard operating procedures, and the time to positive culture was registered. Positive blood cultures were sub-cultured on solid media (37 °C, 5% CO_2_) and incubated overnight to form individual colonies. CSF and BALF cultures were obtained by plating a 200-μl sample on blood agar plates and cultured in an aerobic incubator for at least 14 days. Bacterial or fungal species were identified by MALDI-TOF MS, as described above.

#### Flow cytometry assay of M1 binding with various strains of bacteria

To detect the binding of M1 to various strains of *Escherichia coli*, *Klebsiella pneumoniae*, *Pseudomonas aeruginosa*, and *Enterococcus faecalis*, flow cytometric analyses were performed with randomly selected clinical strains of each species. First, 50-μl aliquots of the bacterial suspensions containing approximately 1 × 10^8^ CFU/ml were centrifuged at 9600 × g for 1 min and washed with 500 μl PBS. The pellet was resuspended in PBS (supplemented with 2 mM CaCl_2_) containing either 10 μg/ml purified M1 or no M1. The suspensions were incubated at 37 °C for 30 min and then centrifuged. The bacterial cell pellet was washed and resuspended in 100 μl of PBS containing 1.5 μg/ml FITC-anti-human IgG Fc (ab 239228) (Abcam Inc, Toronto, ON, Canada, C). This mixture was then incubated in the dark at 37 °C for 20 min before the centrifugation and washing of the bacterial cell pellet. Samples were resuspended in 200 μl of PBS and fixed for at least 30 min by adding 200 μl of 2% (wt/vol) paraformaldehyde in PBS. The samples were kept at 4 °C in the dark until analysis on a flow cytometer. The flow cytometric analyses were performed on a BD FAC-SCanto (Beckton Dickinson) equipped with a 488-nm-blue laser and a 633-nm-red laser. Light transmission data from the FITC-illuminated M1-bacteria complexes were collected by a forward scatter (FSC) detector, side scatter (SSC) detector, and APC fluorescence detector. M1 binding was expressed as FITC median fluorescence intensity (MFI) and categorized as positive M1 binding if the signal was above the negative control prepared with bacteria without M1 but stained with FITC-anti-Human IgG Fc antibody.

#### Capture efficiency of M1 beads with simulated rabbit blood

Because we found that most bacteria were killed or phagocytized by whole blood when incubated with fresh whole blood for 1 h, it was difficult to detect the capture efficiency of M1 beads with simulated fresh whole blood. To inhibit the phagocytic function of whole blood, rabbit blood (2 ml) was treated with 2 μM dexamethasone at 37 °C and 5% CO_2_ for 3 h. Then, 10–50 CFU/ml bacteria (5 clinical strains of each species, *Escherichia coli*, *Klebsiella pneumoniae*, *Pseudomonas aeruginosa* or *Enterococcus faecalis*) were added and incubated for 1 h. After that, the whole culture was plated onto a blood agar plate and incubated overnight to verify the inhibition of dexamethasone on the phagocytic function of whole blood. Blood without dexamethasone treatment was used as a negative control. When no colonies grew on the negative control plates and > 70% colonies (compared with total colonies added) grew on the plates with dexamethasone-treated blood, we regarded the inhibition as successful. Enrichment with M1 beads was performed, as described above.

Capture efficiency was assessed by finding the ratio of the recovered colony numbers with M1 beads to the mean average of three colony numbers recovered from the simulated blood samples.

#### M1-qPCR analyses of pathogens in simulated human whole blood

To reveal why some strains had not been captured by M1 beads from patient blood, while their DNA was detected by M1-qPCR, 7× (10^3^–10^4^) CFU of these strains was added to 1 ml of healthy human blood to simulate blood infection. After incubation for 8 h, the whole cultured blood samples were taken out and M1-real-time PCR method was applied, as described above.

#### Data interpretation and statistical analysis

The clinical judgment of infection was based on the following criteria (need at least 2 out of 3 criteria): 1. fever (> 37.5 °C), without any evident cause other than infections; 2. positive imaging results; and 3. elevated serum lactate, C-reactive protein (CRP) or procalcitonin (PCT). The clinical records of the patients and microbial findings were assessed by two independent and blinded senior physicians in infectious diseases.

The detection performances (sensitivity and specificity) of the M1 method were calculated against the standard culture method. The discrepancies between these methods regarding their ability to detect microorganisms were analyzed with Cochran’s Q test (Gretl software ver. 1.9.4.). A *P* value of < 0.05 was regarded as statistically significant.

## Results

### The detection results of M1-method had a high coincidence rate with that of standard method

During this period of study, a total of 223 hematological malignancy patients during their chemotherapy, autologous or allogeneic HSCT phase entered this study. After exclusion of 3 cases for non-infectious fever, 233 episodes of 220 patients in total were analyzed, including 217 blood samples and 16 BALF and CSF samples. At least one microorganism was detected in 73 episodes by either method. In total, 71 samples were identified by the M1 method. For the standard method, 54 samples were identified. The standard method missed 19 findings identified by the M1 method. However, 2 findings of bacteria identified by the clinical method were not detected by the M1 method. The detailed diagnostic performances for these methods are shown in Table 1.

**Table 1:** Diagnostic performance of the M1 method and standard method

	Standard clinic culture method match (no. [100%]) by result	
M1 result	+	-
+	[24 (blood), 13 (BALF/CSF)] [15 (blood) *by M1-qPCR*] (22.0)	19 (blood) (8.1)
-	2 (blood) (0.8)	[160 (blood), 3 (BALF/CSF)] (69.1)

-, negative; +, positive. The analytical sensitivity of M1 method was 96.3%; positive predictive value (*PPV*) was 73.2%; analytical specificity was 89.6%; and the negative predictive value (*NPV*) was 98.8%.

**Fig. 2:**
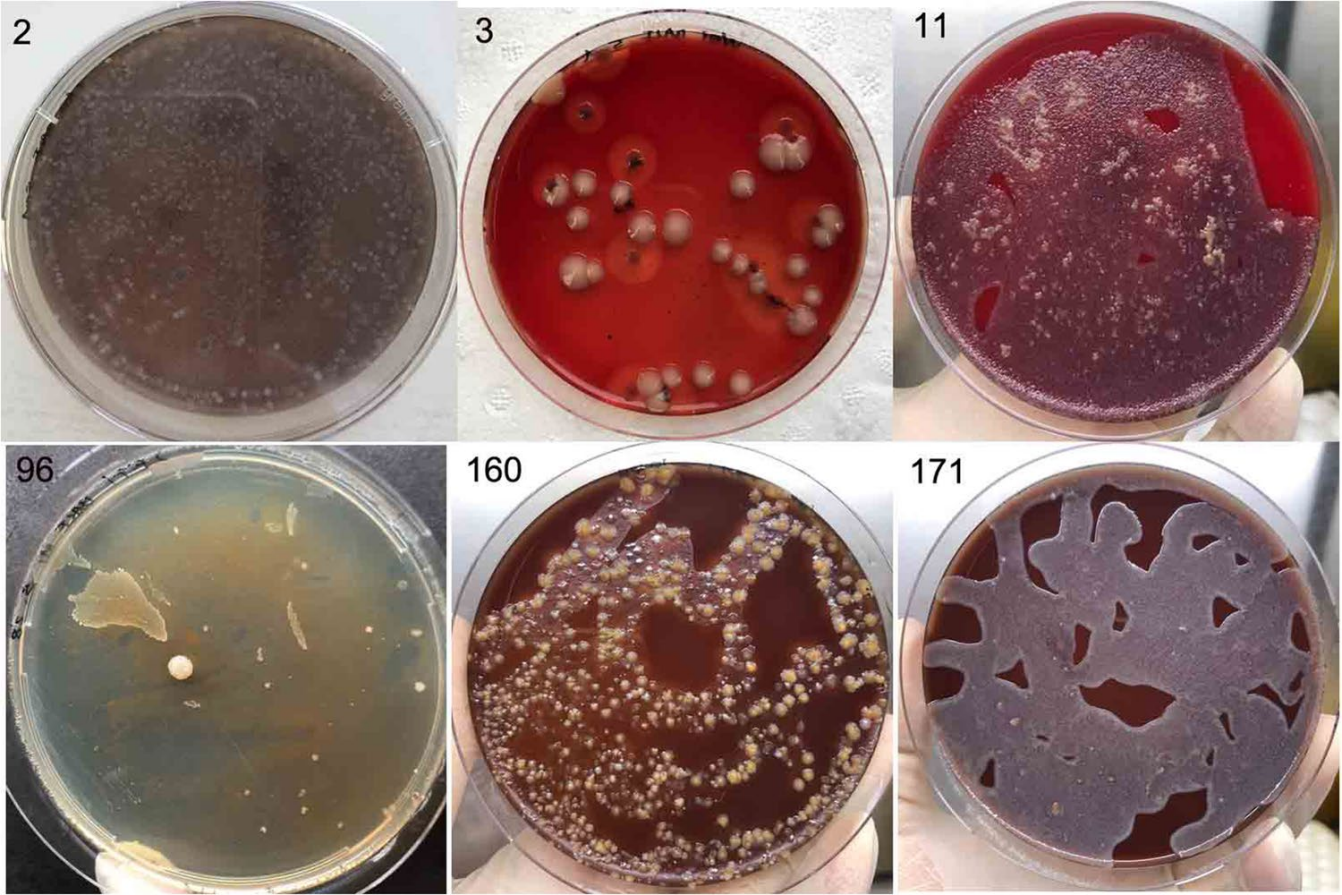
Multiple pathogens cultured by M1-DC in BALF of patients. 2. *S. epidermidis* and *R. mannitolilytica* cultured from number 2 sample; 3. *P. aeruginosa* and *K. pneumoniae* from sample 3; 11, *A. flavus*/*A. oryzae* and *S. maltophilia* from sample 11; 96, *S. pneumoniae* and *R. mucilaginosa* from sample 96; 160, *N. cinerea*, *A. oris*, *N. subflava*, and *R. mucilaginosa* from sample 160; 171, *P. aeruginosa* and *E. cloacae* from sample 171

Significantly, the turn-around time of M1-method was significantly shorter than the standard culture method (Table 2). In total, there were 52 samples which were concordant positive by clinical culture and the M1 method, including 24 blood samples positive for both clinical culture and M1-DC and 15 blood samples positive for both clinical culture and M1-qPCR but negative for M1-DC **(**Table 2**)**. Among the thirteen BALF/CSF samples, multiple pathogens were cultured by M1-DC in about half of these samples (Fig. 2); while standard clinic method only detected one in each sample (Table 3).

**Table 2:** Number of bacteria/fungi detected with the S-BC and M1-method in the blood of patients with BSI

Bacteria species recovered	S-BC	M1-method		
		M1-DC	M1-qPCR	Total
*E. coli*	12	8	4	12
*K. pneumoniae*	8	4	4	8
*P. aeruginosa*	7	2	5	7
*S. epidermidis*	3	3	_	3
*S. aureus*	3	3	_	3
*E. cloacae*	3	3	_	3
*E. faecalis*	2	_	2	2
*C. albicans*	1	1	_	1
Total	39	24	15	39
TTSI (h)	30–96	6–50	3–3.5	

*RN* recovered numbers, *S-BC* clinical culture, *M1-DC M1* enrichment followed by directed culture, *M1-qPCR M1* enrichment followed by *qPCR*, *TTSI* time to species identification, *S. epidermidis Staphylococcus epidermidis*, *S. aureus Staphylococcus aureus*, *E. cloaca Enterobacter cloacae*, *C. albicans Candida albicans*

**Table 3:** Pathogen detected by M1 method in BALF and CSF

Patient number (sample type)	M1 method			Clinical detection	
	DC	qPCR		Culture	Other^a^
		Bacteria	Fungi		
2 (BALF)	*S. epidermidis* ***R. mannitolilytica***	*-*	*-*	*S. epidermidis*	*-*
3 (BALF)	*P. aeruginosa* ***K. pneumoniae***	*-*	*-*	*P. aeruginosa*	
11 (BALF)	*A. flavus/A. oryzae* ***S. maltophilia***	*-*	*Asp*	*A. flavus/A. oryzae*	*-*
73 (BALF)	*S. epidermidis*	*-*	*-*	*S. epidermidis*	*-*
74 (BALF)	*H. parainfluenzae*	*-*	*P. jirovec-ii*	*H. parainfluenzae*	*P. jiroveciia*
87 (BALF)	*K. pneumoniae*	*-*	*-*	*K. pneumoniae*	*-*
89 (BALF)	*C. albicans*	*-*	*Candida* spp.	*C. albicans*	*-*
96 (BALF)	*S. pneumoniae* ***R. mucilaginosa***	*-*	*-*	*S. pneumoniae*	*-*
160 (BALF)	*N. cinerea* *A. oris* ***N. subflava*** ***R. mucilaginosa***	*-*	*-*	*N. cinerea* *A. oris*	*-*
171 (BALF)	*P. aeruginosa* ***E. cloacae***	*-*	*-*	*P. aeruginosa*	*-*
174 (BALF)	*K. pneumoniae*	*-*	*-*	*K. pneumoniae*	*-*
185 (BALF)	*P. aeruginosa*	*-*	*-*	*P. aeruginosa*	*-*
4 (CSF)	*M. tuberculosis*	*M. tuberculosis*	*-*	*M. tuberculosis*	

*S. epidermidis Staphylococcus epidermidis*, *R. mannitolilytica Ralstonia mannitolilytica*, *P. aeruginosa Pseudomonas aeruginosa*, *K. pneumoniae Klebsiella pneumoniae*, *A. flavus Aspergillus flavus*, *A. oryzae Aspergillus oryzae*, *S. maltophilia Stenotrophomonas maltophilia*, *H. parainfluenzae Hemophilus parainfluenzae*, *P. jirovecii Pneumocystis jirovecii*, *C. albicans Candida albicans*, *S. pneumoniae Streptococcus pneumoniae*, *R. mucilaginosa Rothia mucilaginosa*, *N. cinerea Neisseria Cinerea*, *A. oris Aspergillus oris*, *N. subflava Neisseria subflava*, *E. cloacae*, *Enterobacter cloacae*, *M. tuberculosis Mycobacterium tuberculosis*

^a^Microscopic examination

### M1 method detected many pathogens which had not been found by standard clinic method

**Fig. 3:**
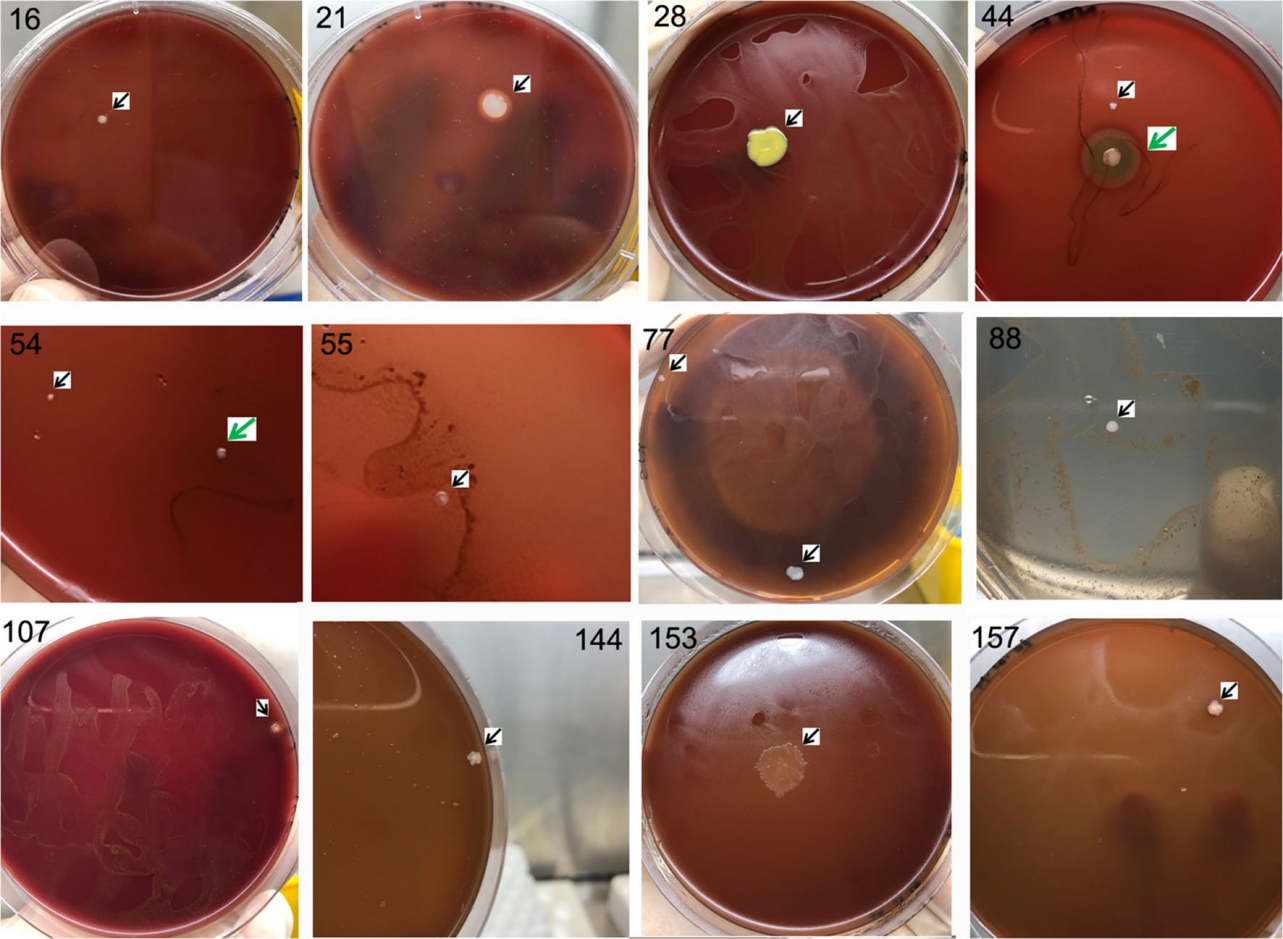
Some pathogens cultured by M1-DC but not by standard blood culture. 16, *C. beijerinckii* cultured from number 16 sample; 21, *S. warnerii* from sample 21; 28, *A. indicus* from sample 28; 44, *B. thuringiensis* (green arrow) and *S. epidermidis* (black arrow) from sample 44; 54, *B. clausii* (green arrow), and *C. flavigene* (black arrow) from sample 54; 55, *Arthrobacter* sp. from sample 55; 77, *S. warnerii* from sample 77; 88, *A. pilosum* from sample 88; 107, *Clostridium* spp. from sample 107; 144, *C. tuberculostearicum* from sample 144; 153, *C. tertium* from sample 153; 157, *K. schroeteri* from sample 157

Nineteen samples missed by the clinical culture method but detected by M1-DC were further identified by qPCR or PCR plus sequencing with patient whole blood. All these patients had clinical data supporting bacterial/fungal infection (Table 4). Some primary culture pictures by M1-DC are shown in Fig. 3.

**Table 4:** Bacteria in patient blood detected by M1-DC or M1-qPCR

Sample number (sample type)	M1 method		Clinic data supporting bacteremia
	DC	qPCR/PCR	
16 (blood)	*C. beijerinckii*	A	Fever, CRP↑, PCT↑, diarrhea, and pneumonia verified by chest CT
20 (blood)	*S. hominis*	-	High fever, acute infective lymphadenitis, CRP↑, and antibiotic treatment effective
21 (blood)	*S. warnerii*	-	Fever and perianal bacterial infection
28 (blood)	*A. indicus*	B	Fever, CRP↑, PCT↑, rigor, diarrhea, abdominal pain, and antibiotic treatment effective
44 (blood)	*B. thuringiensis*; *S. epidermidis*	B	Fever, CRP↑, and diarrhea
47 (blood)	*S. longispora*	B	Cellulitis in right eye frame and left thorax
49 (blood)	*Nonomuraea* sp.	B	Fever, headache, diarrhea, and intestinal infection
53 (blood)	*Saccharithrix* sp.	B	Fever and rigor
54 (blood)	*B. clausii*, *C. flavigene*	A B	Fever, CRP↑, and PCT↑
55 (blood)	*Arthrobacter* sp.	B	Perianal bacterial infection, fever, and antibiotic treatment effective
77 (blood)	*S. warnerii*	-	Pneumonia verified by chest CT
88 (blood)	*A. pilosum*	B	Hemoptysis, pneumonia verified by chest CT
104 (blood)	*C. amylolyticum*	B	High fever, CRP↑, and antibiotic treatment effective
107 (blood)	*Clostridium* spp.	B	High fever, CRP↑, and antibiotic treatment effective
126 (blood)	*C. mucifaciens*	N	Intestinal bacterial infection
144 (blood)	*C. tuberculostearicum*	N	Pneumonia verified by chest CT and bronchoscope
153 (blood)	*C. tertium*	N	High fever and rigor
157 (blood)	*K. schroeteri*	B	PCT↑, rigor, pneumonia with cough, and moist rales
203 (blood)	*C. amylolyticum*	B	Fever, sore throat, upper respiratory tract infection, diarrhea, and antibiotic treatment effective

A, confirmed by real-time PCR; B, confirmed by PCR amplification with specific primer pairs and sequenced; −, negative with either qPCR or PCR; N, not detected for scared of nucleic acid

*A. indicus Agromyces indicus*, *A. pilosum Acremonium pilosum*, *B. clausii Bacillus clausii*, *B. thuringiensis Bacillus thuringiensis*, *C. amylolyticum Clostridium amylolyticum*, *C. beijerinckii Clostridium beijerinckii*, *C. flavigene Cellulomonas flavigene*, *C. mucifaciens Corynebacterium mucifaciens*, *C. tertium Clostridium tertium*, *C. tuberculostearicum Corynebacterium tuberculostearicum*, *K. schroeteri Kytococcus schroeteri*, *S. hominis Staphylococcus hominis*, *S. longispora Saccharothrix longispora*, *S. warnerii Staphylococcus warnerii*.

### M1 enrichment could identify the bacteria directly or indirectly by detecting bacterial DNA using qPCR

**Fig. 4:**
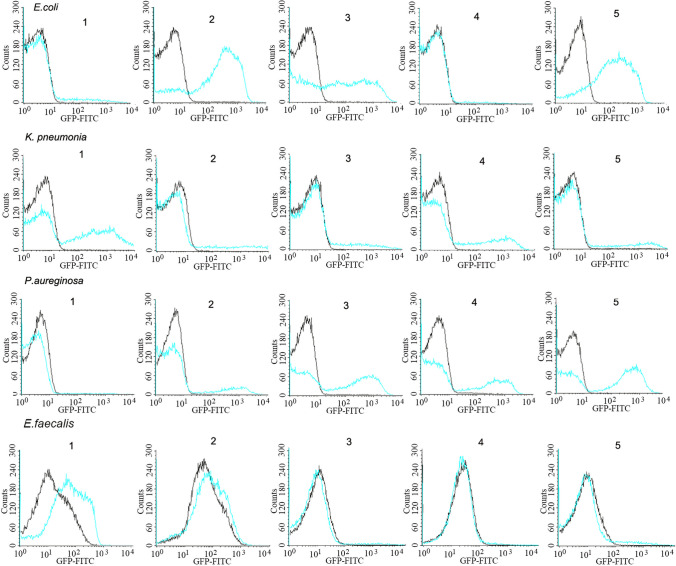
A representative flow cytometric overlay showing the binding of M1 with 5 randomly selected clinical strains of each species. Black lines represent the control samples incubated without M1; blue lines represent samples with 10 μg/ml M1

Flow cytometric analyses revealed that M1 did bind with some randomly selected clinical strains with variable capability (Fig. 4). The binding capability of M1 described as the mean fluorescence intensity (MFI) changed from 3.99 to 363.80 for *E. coli*, 13.48 to 467.28 for *K. pneumonia*, 8.36 to 298.16 for *P. aeruginosa*, and 8.87 to 50.48 for *E. faecalis*. Further studies with simulated blood samples (10–50 CFU/ml) demonstrated that those strains with higher MFI always had higher capture efficiencies with M1 beads than the strains with lower MFI (Table 5).

**Table 5:** The MFI, capture efficiency (*CF*), and qPCR detection results (*CT* value) of 5 randomly selected strains of *E. coli*, *K. pneumoniae*, *P. aeruginosa*, and *E. faecalis*

Strain	Testing Index	*E. coli*	*K. pneumoniae*	*P. aeruginosa*	*E. faecalis*
1	MFI	5.14 ± 0.15	467.28 ± 38.21	8.36 ± 2.73	50.48 ± 7.66
	CF	11.05 ± 2.62%	48.38 ± 9.36%	5.12 ± 3.80%	23.33 ± 4.85%
	CT	34.21 ± 3.63	33.28 ± 2.15	34.21 ± 2.22	35.52 ± 4.28
2	MFI	363.80 ± 27.63	28.61 ± 5.97	151.71 ± 28.65	13.15 ± 5.54
	CF	45.31 ± 6.72%	5.62 ± 4.28%	28.69 ± 17.23%	9.28 ± 4.89%
	CT	33.67 ± 4.28	35.63 ± 2.67	33.14 ± 3.27	34.76 ± 5.38
3	MFI	222.23 ± 38.96	13.48 ± 4.86	278.63 ± 38.82	11.76 ± 3.56
	CF	35.15 ± 7.62%	7.35 ± 3.69%	42.08 ± 17.21%	6.43 ± 4.47%
	CT	35.62 ± 3.32	36.31 ± 2.90	35.67 ± 3.20	36.36 ± 4.37
4	MFI	3.99 ± 1.22	134.60 ± 57.82	200.33 ± 45.79	9.66 ± 2.25
	CF	8.21 ± 1.57%	22.63 ± 3.27%	33.21 ± 15.62%	7.65 ± 3.86%
	CT	34.28 ± 4.89	34.61 ± 3.28	34.80 ± 2.15	35.44 ± 3.36
5	MFI	275.55 ± 40.52	110.17 ± 29.37	298.16 ± 52.28	8.87 ± 3.21
	CF	36.23 ± 7.28%	20.78 ± 4.58%	38.71 ± 10.62%	6.26 ± 4.76%
	CT	33.67 ± 2.36	36.28 ± 3.6	33.75 ± 3.12	35.04 ± 4.33

Data are the *means* ± *SD* of assays performed over 5–6 repetitions. Those strains marked with shadows had higher MFI and higher capture efficiencies with M1 beads.

*MFI* mean fluorescence intensity, *CF* capture efficiency, *qPCR* detection was performed after M1 bead enrichment with simulated human blood samples infected with 7× (10^3^–10^4^) CFU of those strains of *E. coli*, *K. pneumoniae*, *P. aeruginosa*, and *E. faecalis* for 8 h.

To reveal why some strains had not been cultured by M1 beads from patient blood, while their DNA was detected by M1-qPCR, 7× (10^3^–10^4^) CFU of these strains was added to 1 ml of human blood to simulate blood infection. After incubation for 8 h, almost all these bacterial DNA could be detected by qPCR from the M1 bead-captured samples. The *CT* values showed no significant differences between strains with different MFI binding values (Table 5).

## Discussion

In this study, we assayed the application of M1 bead enrichment in the detection of possible pathogens in hematological malignancy patients. Although it was not a strictly etiological study of infection, this M1 method added diagnostic value by supporting the detection of clinically relevant microbes missed by the standard method. Especially for BALF and CSF samples, the results of the M1 method corresponded well to those of the standard method. Most significantly, M1 method saved as least 24 h compared with standard culture method, which is probably lifesaving for HSCT patients with severe acute infection.

The heterogeneity of M1 beads binding was based on the broad binding spectrum of MBL, which was able to bind with various microbial carbohydrate structures (Neth et al. 2000). As we demonstrated before, M1 was a polymer with 3 homodimers (i.e., 6 CRDs) in native state, which had much higher binding capacity than usual homodimer forms constructed with Fc-fused monomers, even higher than that of tetramer form of MBL expressed in vitro with full length of *MBL* gene (Chen et al. 2020). Since a more proper array of CRDs would more easily interact with the sugar patterns on the microbial surface (Dominic et al. 2001), we suspected that the binding ability was determined not only by the degree of CRD oligomerization but also by the spatial structure formed by clustering of CRDs, while M1 has a more proper spatial structure of CRDs to bind with more pathogens. This might explain why native MBL or recombinant MBL of homodimers did not bind with some species of bacteria such as *P. aeruginosa* and *E. faecalis* (Dominic et al. 2001; Seiler et al. 2019), while M1 was shown to bind with some strains of these species. Hence, M1 beads has a great potential for impacting the broad application in clinical microbial detection.

Interestingly, some strains of *E. coli*, *K. pneumoniae*, *P. aeruginosa*, and *E. faecalis* had not been cultured directly by M1-DC. However, their nucleic acid was positive by M1-qPCR. Our study also showed that those bacteria (with low binding capacity with M1) could also be detected by M1-qPCR after cultured with healthy human blood for 8 h, when bacteria were killed and fragmented by blood ingredients such as complements and bacteriolysin. We speculated that .these inactive bacteria could be well captured by M1 beads since the binding to MBL was increased for those inactive and fragmented bacteria (Seiler et al. 2019). Then, these bacteria could not be cultured by M1-DC but detected by M1-qPCR. Recently, PANoptosis (necroptosis, pyroptosis, and apoptosis) of host cells was found to occur to protect against most acute bacterial pathogens, even non-pathogenic or opportunistic bacteria. These dying cells would expose a large variety of intracellular molecules on the cell surface (Bertheloot et al. 2021; Place et al. 2021), while MBL could bind to these molecules by their globular heads, linking the dying cells to phagocytes (Casals et al. 2019). We speculated that another explanation was M1 beads (with CRD) might bind with dying leukocytes infected with pathogens. Then, M1 method could identify the bacteria directly or indirectly by detecting bacterial DNA using qPCR. The M1 method could probably be applied to detect almost all pathogens with nucleic acid method.

Culture by classifying all the findings is judged as clinically relevant as “true positive” (Lucignano et al. 2011). While in this study, we compared the results of M1 method with standard clinic culture since it is still the gold standard at present. Our results showed that the analytical sensitivity of M1 method reached 96.3% compared with the standard clinic culture method. Furthermore, the clinical utility of M1 method is also highlighted by the time-consuming nature of the standard culture method. However, compared with the standard culture method, the PPV of M1 method was only 73.2% and analytical specificity only 89.6%. We contributed it to the fact that many bacteria could only be detected by M1 method.

Intriguingly, many microorganisms cultured using the M1 method were rare bacteria. However, microorganisms such as *B. thuringiensis*, *C. mucifaciens*, *C. tuberculostearicum*, *Clostridium* spp., *K. schroeteri*, *B. clausii*, *Arthrobacter* spp., and *Acremonium* spp. were all reported as causing pathogens of systemic infection (Hsu et al. 1998; Lau et al. 2002; Benjamin et al. 2006; Yamazaki et al. 2006; Kuroki et al. 2009; Amaraneni et al. 2015; Princess et al. 2020; Yamamuro et al. 2021). *Nonomuraea* sp. was reported as a possible pathogen in pneumonia (Truong et al. 2019). However, the clinical significance of blood infection with *Saccharothrix* spp. cultured by M1-DC in two patients was analyzed in detail in our other paper (Zheng et al. 2022). We suspected the reasons that these microorganisms could not be cultured by clinical blood culture are the following: ingredients like antibiotics that inhibit bacterial growth were present in blood but removed in M1-DC; some fastidious bacteria like actinomycetes, which were always negative with standard blood culture, might be cultured by M1-DC through enrichment and plated on agar plates directly. However, we recognize that it remains challenging to determine the etiological significance of all these organisms detected in blood, and further studies should be conducted.

The challenge of determining the etiological significance of detected organisms is a concern not only in bacterial culture but also in nucleic acid detection (Peri et al. 2022). In this study, the choice of organisms for qPCR detection was based on expected pathogenicity and clinical presentation as previously described in hematological malignancy patients, such as *Mycobacterium tuberculosis* (TB), *non-tuberculosis mycobacteria* (NTB), *P. jirovecii*, *M. pneumoniae*, and fungi (Sahin et al. 2016). Our study indicated that M1-based qPCR had significantly higher sensitivity than the standard qPCR method, most likely by decreasing the abundance of human nucleic acids in whole blood. Also, other species could be included in the M1-qPCR method to expand the pathogen detection spectrum in the future.

At present, molecular detection methods, such as PCR, are becoming promising alternatives to standard blood culture methods due to their time-saving advantages. However, the sensitivities of molecular methods are not always sufficient, especially for blood samples. Thus, pretreatment, including pathogen enrichment, is always necessary even for mNGS (Dubourg et al. 2019). It was promising that samples including blood, CSF and BALF treated with M1 beads had higher positive rates and lower *CT* values than those that were not treated. However, we also acknowledged that M1 bead enrichment method, like other enrichment methods, has the limitations of additional hands-on time, cost, and ease of batching, while all these limitations could be conquered by application of automatic instruments.

In a word, our study demonstrated M1 method can identify the bacteria directly or indirectly by detecting bacterial DNA using qPCR, even when the bacteria are not recoverable by culture, M1 bead enrichment increased the detection efficiency of most pathogens in hematological malignancy patients. With some modifications and supplements, this method might also be a promising alternative to detect all pathogens during sepsis. Further studies should be performed to confirm the binding of M1 beads with infected host cells.

## Supplementary information

Supplementary file 1

## Data Availability

The data generated during the current study are available from the corresponding author on reasonable request.
