# Supplementary material for: Recombinant mannan-binding lectin magnetic beads increase pathogen detection in immunocompromised patients
Source: Appl Microbiol Biotechnol. 2024 Feb 3;108:193. doi: 10.1007/s00253-024-13019-3 (PMC10838228; doi:10.1007/s00253-024-13019-3)
Supplement: Supplementary file 1 [file 253_2024_13019_MOESM1_ESM.pdf]

Applied Microbiology and Biotechnology

Recombinant mannan-binding lectin magnetic beads increase pathogen detecti in immunocompromised patients

Chen Xiao-Ping[1¶], Zheng Hao[1¶], Feng Ru-Li[3], Lu Jin-Xing[1], Dong Yu-Jun[2*], Liang Ze-Yin[2*]

Affiliations:

1. National Institute for Communicable Disease Control and Prevention, Chinese Center for Disease Control and Prevention, Beijing, China

2. Department of Hematology, Peking University First Hospital, Beijing, China

3. Clinical Laboratory of Peking University First Hospital, Beijing, China

[*] Corresponding author Dong Yu-Jun XiShiKu Street 8, XiCheng District, Beijing, China; 86-10-83572211; Email: dongyj@hsc.pku.edu.cn; Liang Ze-Yin XiShiKu Street 8, XiCheng District, Beijing, China; 86-10-83572211; Email: : walzyaw@163.com

[¶] These authors contributed equally to this work. The authors have declared that no conflict of interest exists.

Supplementary Table 1. Real-time PCR primer probes.

Pathogen species	Upper primer (5'-3')	Down primer (5'-3')	Probe (5'-3')	Ref
S.epidermidis	ATATTTCAGATGCGGCATTAG	CAATTCTTTTTCGAGTTGAGTGAT	FAM-ACATTGCACAGTCTGCGATTAGTC-BHQ1	Yang et al. 2019
E.coli	CTTCGAGACGGGCTACGC	GAACGCTTATGGTCACGGTCT	FAM-CGTCAGCATCGGGGAGCGTT-BHQ1	
E.faecium	TTCTTTGCTTTATCCGATGT	CGGTTTTCGCTTTTGTAAT	FAM-ACTAGAACCCATATTCGCC-BHQ1	Oeser et al. 2020
B.thuringiensis	GGGCATCAAATAATGGCTTC	GCTGCATTTCCCATAGTTCC	FAM-CCTGCATTTCCCATAGTTCC-BHQ1	Sedlackova et al. 2017
S. homonis	TAGATGGATCTGAAACAGTAGTAT	CCTTCAACAATACCAAATTCGTC	FAM-AGGTGCTTCATGTACTACAAACTCATTG-BHQ1	Kilic et al. 2011
C.beijerinckii	ATGACGTTAATGCAATGGGAATT	CGTGTCACCTTCCGATGTTTT	FAM-TAGAATTTTATGAGGATGGAAGC-BHQ1	Morandi et al. 2015
B.clausii	AATTTTTACCGCCCCTCAAG	ACTTTTGGAACATGCCGAAC	FAM-TGCCAGGCAGTGGGCGATGG-BHQ1	Yang et al. 2019
P.aeruginosa	TCAACCTGAAGGAGGATT	CCTTCTTGGCCTTGTCGAG	FAM-CTGTCGCTGCTGTCGTCGCTT-BHQ1	Zheng et al. 2017
M.tuberculosis	GCTCGCGTAGGGCTT	CCCTTGCCACCACGA	CY5-ATTCACGAGGTTCAGCGTCGA-BHQ2	Abdeldaim et al. 2016
NTB	CAGCAYCCCGGGTGAC	GACCATCCATCTGGRYACA	FAM-CCGCTYGAGGYACCCG-BHQ1	
P. jirovecii	GAATGCAAATCYTTACAGACAACAG	AAATCATGAACGAAATAACCATTGC	FAM-CAACCTGAACTTAGTAGCGCAAGGCCAA-3-BHQ1	Sasso et al. 2016
M. pneumoniae	CGCTTACTGTACGATGAACTTGAAA	AGGGTGTGAAGAGTTGCAAGTCT	FAM-CAACCTGAACTTAGTAGCGCAAGGCCAA-3-BHQ1	Schmitt et al. 2013
Fungia	TTGGTGGAGTGATTTGTCTGCT	TCTAAGGGCATCACAGACCTG	Hex-TTAACCTACTAAATAGTGCTGCTAGC-TAMRA(yeast fungi)	Gosiewski et al. 2014

		Cy5-TCGGCCCTTAAATAGCCCGGTCCGC(filamentous fungi)

NTB, non-tuberculous mycobacteria. *S.epidermidis*, *E.coli*, *E.faecium*, *B.thuringiensis*, *S. homonis*, *C.beijerinckii*, *B.clausii*, *P.aeruginosa* were selected to detect for these bacteria were positive for M1-DC, but negative for standard blood culture. Then real-time PCR was further applied to confirm the results. *M.tuberculosis*, NTB, *P. jirovecii*, *M. pneumoniae* and Fungia were chosen for their expected pathogenicity and clinical presentation as previously described in hematological malignancy patients.

Supplementary Table 2. Primers amplifying specific bacteria

**Bacteria species**	**Upper primer(5'-3')**	**Down primer(5'-3')**	**Length (bp)**	**References**
A.indicus	AGA CAC GGC CCA GAC TCC TAC G	CTC CCC ACG CTT TCG CTC CTC A	432	This study
Arhrobacter sp	CGC CGC GTG AGG GAT GAA TG	GGC GCG GAA AAC GTG GAA TGT C	441	This study
Cellulomonas sp	CTC GCG GCC TAT CAG CTT GTT GGT	CTG CCT TCG CCA TCG GTG TTC CT	481	This study
Clostridium sp	TAC GGG AGG CAG CAG TGG GGA ATA	TGT TAA CGG CGG CAC GGA GGA AT	500	This study
C.amylolyticum	CGC CGC GTG AGT GAT GAA GG	GGC GGC ACG GAA GGA GTC G	438	This study
K.schroeteri	CCA AGG CGA CGA CGG GTA GC	CTT CGC CAT CGG TGT TCC TCC TGA	439	This study
Nonomuraea sp.	AGA CAC GGC CCA GAC TCC TAC G	CTC CCC ACG CTT TCG CTC CTC AG	429	This study
S.warneri	CGG CGG ACG GGT GAG TAA CA	CGT GGG CTT TCA CAT CAG ACT T	522	This study

K.schroeteri, Kytococcus schroeteri;A.indicus, Agromyces indicus;C.amylolyticum, Clostridium amylolyticum;

References

Gosiewski T, Jurkiewicz-Badacz D, Sroka A, Brzychczy-Włoch M, Bulanda M (2014) A novel, nested, multiplex, real-time PCR for detection of bacteria and fungi in blood. BMC Microbiol 14: 144. http://doi: 10.1186/1471-2180-14-144.

Kilic A, Basustaoglu AC (2011) Double triplex real-time PCR assay for simultaneous detection of *Staphylococcus aureus*, *Staphylococcus epidermidi*s, *Staphylococcus hominis*, and *Staphylococcus haemolyticus* and determination of their methicillin resistance directly from positive blood culture bottles. Res Microbio. 162:1060-1066. http://doi: 10.1016/j.resmic.2011.07.009.

Morandi S, Cremonesi P, Silvetti T, Castiglioni B, Brasca M (2015) Development of a triplex real-time PCR assay for the simultaneous detection of *Clostridium beijerinckii*, *Clostridium sporogenes* and *Clostridium tyrobutyricum* in milk. Anaerobe 34: 44-49. http://doi: 10.1016/j.anaerobe.2015.04.005.

Oeser C, Pond M, Butcher P, Bedford Russell A, Henneke P, Laing K, Planche T, Heath PT, Harris K.(2020) PCR for the detection of pathogens in neonatal early onset sepsis. PLoS One 15: e0226817.http://doi: 10.1371/journal.pone.0226817.

Sasso M, Chastang-Dumas E, Bastide S, Alonso S, Lechiche C, Bourgeois N, Lachaud L (2016) Performances of four real-time PCR assays for diagnosis of *Pneumocystis jirovecii* pneumonia. J Clin Microbiol 54: 625-630. http://doi: 10.1128/JCM.02876-15.

Sedlackova V, Dziedzinska R, Babak V, Kralik P (2017) The detection and quantification of *Bacillus thuringiensis* spores from soil and swabs using quantitative PCR as a model system for routine diagnostics of *Bacillus anthracis*. J Appl Microbiol. 123: 116-123. http://doi: 10.1111/jam.13445.

Schmitt BH, Sloan LM, Patel R (2013) Real-time PCR detection of *Mycoplasma pneumoniae* in respiratory specimens. Diagn Microbiol Infect Dis 77: 202-205. doi: 10.1016/j.diagmicrobio.2013.07.016.

Yang J, Qi XM, Wu YG (2019) The application analysis of multiplex real-time polymerase chain reaction assays for detection of pathogenic bacterium in peritoneal dialysis-associated

peritonitis.Blood Purif 47: 337-345.https://doi: 10.1159/000495780.

Zheng h, Li WG, Yang HY, Wu Y, Che J, Chen XP, Ju JX (2017) Establishment of a multiplex RT-PCR assay for common pathogens causing sepsis. Disease Surveillance 9: 752-756.

Abdeldaim G, Svensson E, Blomberg J, Herrmann B (2016) Duplex detection of the *Mycobacterium tuberculosis* complex and medically important non-tuberculosis mycobacteria by real-time PCR based on the rnpB gene. APMIS 124: 991-995. http://doi: 10.1111/apm.12598.
